# Cholesterol Synthesis Is Important for Breast Cancer Cell Tumor Sphere Formation and Invasion

**DOI:** 10.3390/biomedicines10081908

**Published:** 2022-08-06

**Authors:** Hee Yeon Kim, Sung Jin Bae, Ji-Woong Choi, Suji Han, Seung-Hyun Bae, Jae-Ho Cheong, Hyonchol Jang

**Affiliations:** 1 Anticancer Resistance Branch, Division of Rare and Refractory Cancer, National Cancer Center, Research Institute, Goyang 10408, Korea; 2 Department of Biochemistry and Molecular Biology, Yonsei University College of Medicine, Seoul 03722, Korea; 3 Wide River Institute of Immunology, Seoul National University, Hongcheon 25159, Korea; 4 Department of Surgery, Yonsei University College of Medicine, Seoul 03722, Korea; 5 Department of Cancer Biomedical Science, National Cancer Center Graduate School of Cancer Science and Policy, Goyang 10408, Korea

**Keywords:** cholesterol, breast cancer, tumor sphere, invasion, malignancy

## Abstract

Breast cancer has a high risk of recurrence and distant metastasis after remission. Controlling distant metastasis is important for reducing breast cancer mortality, but accomplishing this goal remains elusive. In this study, we investigated the molecular pathways underlying metastasis using cells that mimic the breast cancer distant metastasis process. HCC1143 breast cancer cells were cultured under two-dimensional (2D)-adherent, tumor sphere (TS), and reattached (ReA) culture conditions to mimic primary tumors, circulating tumor cells, and metastasized tumors, respectively. ReA cells demonstrated increased TS formation and enhanced invasion capacity compared to the original 2D-cultured parental cells. In addition, ReA cells had a higher frequency of ESA^+^CD44^+^CD24^−^ population, which represents a stem-cell-like cell population. RNA sequencing identified the cholesterol synthesis pathway as one of the most significantly increased pathways in TS and ReA cells compared to parental cells, which was verified by measuring intracellular cholesterol levels. Furthermore, the pharmacological inhibition of the cholesterol synthesis pathway decreased the ability of cancer cells to form TSs and invade. Our results suggest that the cholesterol synthesis pathway plays an important role in the distant metastasis of breast cancer cells by augmenting TS formation and invasion capacity.

## 1. Introduction

Although the 5-year survival rate for breast cancer is as high as 90%, breast cancer is the second leading cause of cancer death among women [1]. After 5 years, a significant number of breast cancer patients recur, and the prognosis of these patients is extremely poor [2]. The high recurrence rate primarily results from metastasis, and a gene signature associated with invasiveness has been found to be highly associated with metastasis-free survival and overall survival [3,4]. Therefore, there is a need to prevent distant metastasis to reduce breast cancer mortality.

Distant metastasis results from cancer spreading from its original site to a different organ through lymph nodes or the bloodstream [5]. In the case of breast cancer cells, they spread to other sites of the body, such as bone [6], brain [7], and lung [8] tissues, even after remission. Circulating tumor cells (CTCs) that have left the primary site must settle and adapt to the new cellular environment via extravasation [9,10]. To control distant metastasis, it is necessary to study essential factors or signaling pathways in CTCs circulating in the bloodstream and metastasized tumors adapted to a new environment [11]. However, methods to study this process in vitro are limited. Tumor spheres (TSs) may partially reflect the properties of CTCs that remain viable in the bloodstream after losing their attachment to the basement membrane [12]. Reattached (ReA) cells obtained by reattaching the TSs can mimic distant metastases to some extent. It is thought that this model can partially mimic the distant metastasis process.

Cholesterol metabolism is associated with breast cancer metastasis [13,14,15,16,17]. In a mouse model of breast cancer, in which mice were fed a high-fat diet, serum cholesterol levels, tumorigenicity, and metastasis to lung tissues increased [13]. Cholesterol biosynthesis-related genes and metabolites increase the metastatic potential of breast cancer [14,15]. Additionally, cholesterol metabolism is increased in breast cancer TSs [16,17] and influences breast cancer patient prognoses [17]. These results suggest a close relationship between breast cancer metastasis, TSs, and cholesterol metabolism. However, there is still a lack of conclusive evidence as to whether cholesterol metabolism plays an important role in tumors that recur through distant metastasis.

Here, we investigated the major signaling pathways involved in breast cancer metastasis using an in vitro model that mimics the distant metastatic process. Our results show that the intracellular cholesterol was elevated in TSs and ReA cells. Inhibition of the cholesterol synthesis pathway hampered TS formation and reduced invasion capacity in breast cancer, suggesting that it is important for distant metastasis.

## 2. Materials and Methods

### 2.1. Cell Culture and Chemical Materials

Human breast cancer cell lines HCC1143, BT-549, MDA-MB-231, MDA-MB-453, and MCF7 were purchased from the Korean Cell Line Bank. Breast cancer cells were cultured in 2D in RPMI complete medium at 37 °C in humidified incubators containing 5% CO_2_, except for MDA-MB-231 cells, which were cultured in DMEM complete medium. RPMI complete medium consists of RPMI (#SH30027.01; HyClone, Logan, UT, USA) supplemented with 10% heat-inactivated fetal bovine serum (FBS, #SH30084.03; HyClone) and 1% penicillin/streptomycin (#15140-122; Invitrogen, San Diego, CA, USA). DMEM complete medium is identical to RPMI complete medium, except that DMEM (#SH30243.01; HyClone) is used instead of RPMI. TS cells were cultured according to the ex vivo CTC culture method described previously [12], with some modifications [18]. Briefly, TS culture medium consisted of RPMI-medium-supplemented 1× B27 (#17504-044; Invitrogen), 20 ng/mL basic fibroblast growth factor (#100-18B; PEPROTECH, Cranbury, NJ, USA), 20 ng/mL epidermal growth factor (#E9644; Sigma-Aldrich, St. Louis, MO, USA), 1% penicillin/streptomycin, and Cellmaxin plus (#C3319-020; GenDEPOT, Austin, TX, USA). Single-cell dissociated cells were suspended and cultured on polyhema (#P3932; Sigma-Aldrich)-coated plates for 3–5 days. TS cells were passaged at least five times to distinguish them from cell aggregates and for stabilization. Reattached (ReA) cells were obtained by reattaching TS to the plate. ReA cells were cultured using the same method as the parental cells.

The cells were authenticated and checked for Mycoplasma at the Genomics Core Facility (National Cancer Center, Goyang, Korea), as described previously [19]. Lovastatin (mevinolin; #M2147), alendronate (alendronate sodium trihydrate; #A4978), squalestatin 1 (zaragozic acid A; #Z2626), and IGEPAL^®^ CA-630 (NP-40; #I3021) were purchased from Sigma-Aldrich. Chloroform (#102445) and isopropanol (#109634) were purchased from Merck (Darmstadt, Germany).

### 2.2. Flow Cytometry

Samples were analyzed at the Flow Cytometry Core Facility (National Cancer Center) using a FACS Verse Flow Cytometer (BD Biosciences), as described previously [20]. Cell surface expression of ESA (also known as EPCAM), CD24, and CD44 was evaluated using CD24-PE- (#555428), CD44-APC- (#559942), and ESA-Bv421-specific (#563180) antibodies, all of which were purchased from BD Biosciences (Franklin Lakes, NJ, USA). ESA^+^ populations were first gated, and then CD24^−^ and CD44^+^ populations were quantified using FlowJo (ver. 10.7; Tree Star Inc., Ashland, OR, USA). The ESA^+^CD44^+^CD24^−^ population was considered a cancer-stem-cell-like (or stemness) population.

### 2.3. Tumor Sphere Formation and Quantification

**Figure 1:**
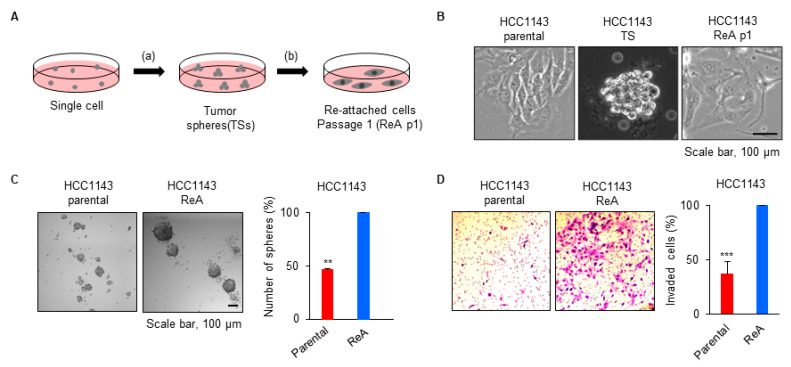
ReA cells after TS culture are more malignant than parental cells. (**A**) A scheme showing the culture methods for HCC1143 ReA cells. (**B**) Bright-field images of cells were taken with Axio Observer Z1 (Carl Zeiss, Jena, Germany). (**C**) Tumor sphere formation assay of parental and ReA cells. The proportion of tumor sphere cells was quantified as the mean ± standard of three independent experiments. Bright-field images of cells were taken with a cytation3 Cell Imaging Multi-Mode Reader. ** *p* < 0.01 relative to parental cells. (**D**) Boyden chamber invasion assay of parental and ReA cells. The proportion of invaded cells was quantified as the mean ± standard deviation of three independent experiments. *** *p* < 0.001 relative to parental cells.

Tumor sphere formation was performed as described previously, with some modifications [18]. Parental, TS, and ReA breast cancer cell lines were seeded in an ultra-low-attachment 96-well plate (#3474; Corning Costar Corp., Cambridge, MA, USA). Cells were seeded at a density of 1 × 10^3^ cells/well in case of Figure 1, and 3 × 10^3^ cells/well in case of Figure 4. TS cells with diameters of more than 100 μm were counted.

### 2.4. Boyden Chamber Invasion Assay

The invasion assay was performed according to a modified version of a method described previously [21]. The upper compartments of 8 mm Transwell chambers (6.5 mm diameter; Corning Costar Corp., Corning, NY, USA) were precoated with 10 mg/mL of Matrigel (Corning Costar Corp.). Equal number of resuspended cells in serum-free RPMI medium were placed in the upper compartments of the Transwell chambers, and the lower compartments were filled with the RPMI complete medium. Breast cancer cells were treated with the indicated concentration of lovastatin for 48 h, and then cells were placed in the upper compartments at a density of 3 × 10^3^ cells/well in case of ReA cells, and 1 × 10^4^ cells/well in case of parental cells. After 16 h, the filters were washed and stained using a Diff-Quik Staining Kit (#38721; Sysmex, Kobe, Japan). Each assay was performed three times independently, and three random fields were analyzed under ×20 magnification for each filter membrane.

### 2.5. Sulforhodamine B (SRB) Assay

Breast cancer cells were treated with the indicated concentration of lovastatin for 48 h, and then cells were seeded at 1 × 10^4^ cells/well in a 96-well plate. The plates were incubated at 37 °C in humidified incubators containing 5% CO_2_. After 16 h, the SRB assay was performed as described previously [22].

### 2.6. RNA Sequencing

Total RNA was extracted with TRIzol (#15596026, Invitrogen) and RNA sequencing was performed by the Macrogen (Seoul, Korea) according to the method described previously [23]. Briefly, total RNA concentration was calculated by Quant-IT RiboGreen (Invitrogen, #R11490). To assess the integrity of the total RNA, samples were run on the TapeStation RNA screentape (Agilent, #5067-5576). Only high-quality RNA preparations, with a RNA integrity number (RIN) greater than 7.0, were used for RNA library construction. A library was independently prepared with 1 μg of total RNA for each sample by Illumina TruSeq Stranded mRNA Sample Prep Kit (Illumina, Inc., San Diego, CA, USA, #RS-122-2101). The libraries were quantified using KAPA Library Quantification kits for Illumina Sequencing platforms according to the qPCR Quantification Protocol Guide (KAPA BIOSYSTEMS, #KK4854) and qualified using the TapeStation D1000 ScreenTape (Agilent Technologies, #5067-5582). Indexed libraries were then submitted to an Illumina HiSeq2500 in case of HCC1143 TS and NovaSeq 6000 in case of HCC1143 ReA, and paired-end (2 × 101 bp) sequencing was performed.

### 2.7. RNA Sequencing Data Analysis

Differentially expressed genes (DEGs) were filtered based on a fold change >2. Genes with transcripts per million values <10 in all samples were excluded from the subsequent analysis. A heatmap of DEGs was created using a MultiExperiment Viewer (The Institute of Genomic Research, Rockville, MD, USA). DEGs were subjected to core analysis using Ingenuity Pathway Analysis (IPA) software (Qiagen, Hilden, Germany), as described previously [23,24].

### 2.8. Cholesterol Quantitation Assay

Lipids from an equal number of parental and ReA cells were extracted using chloroform, isopropanol, and NP-40 at a ratio of 7:11:0.1. Free cholesterol levels were measured using a Cholesterol/Cholesteryl Ester Assay kit (#ab65359; Abcam, Cambridge, UK) according to the manufacturer’s instructions.

### 2.9. Statistical Analysis

Statistical analyses were performed as reported previously [24]. Numerical values are expressed as the mean ± standard deviation, and *p*-values were calculated using the Student’s *t*-test calculator (http://graphpad.com/quickcalcs/) (accessed on 24 July 2022).

## 3. Results

### 3.1. ReA Cells after TS Culture Are More Malignant than Parental Cells

CTCs, which play a significant role in distant metastasis, exist in the bloodstream in a three-dimensional form and extravasate to form tumors at distant sites [10,25]. To mimic this process of settling and adapting to a new site, we reattached TS cells under the same conditions as the parental cells, and they were designated ReA cells (Figure 1A). Passage of the first attached cells was designated p1 (Figure 1B). TSs have been known to have high TS formation and invasion ability [26], and we wanted to investigate whether these characteristics are still maintained in ReA cells. After seeding the same number of cells and observing for 7 days, the ability to form TSs was about two-fold higher in ReA cells than in parental cells (Figure 1C). Similarly, in the Boyden chamber invasion assay, ReA cells had approximately a two-fold higher ability to invade than parental cells (Figure 1D). These results suggest that ReA cells are more aggressive than parental cells, similar to TS cells.

### 3.2. The ReA Cell Population Exhibits an Increased Proportion of ESA^+^CD44^+^CD24^−^ Cells

**Figure 2:**
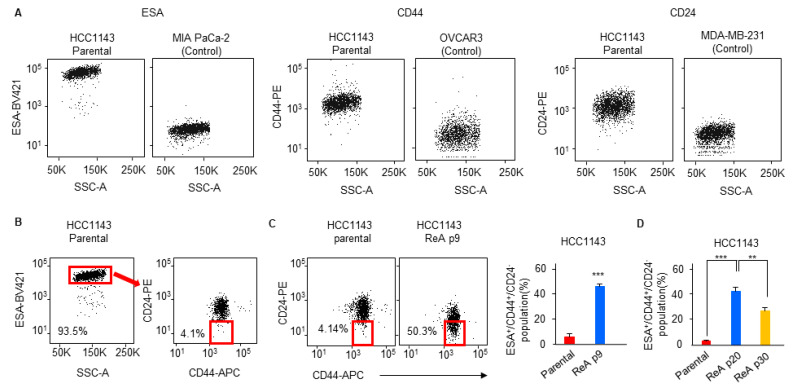
The ReA cell population exhibits an increased proportion of ESA^+^CD44^+^CD24^−^ cells. (**A**) Flow cytometry analysis to optimize conditions for identifying positive or negative cells for the three markers (ESA, CD44, and CD24). (**B**) The ESA^+^ population was gated, and the CD44^+^CD24^−^ population was examined in HCC1143 parental cells. (**C**,**D**) The proportion of ESA^+^CD44^+^CD24^−^ population in ReA cells by passage was examined and quantified using flow cytometry. Values are represented as the mean ± standard deviation of three independent experiments. ** *p* < 0.01, *** *p* < 0.001 relative to parental cells.

In breast cancer, the ESA^+^CD44^+^CD24^−^ population is considered as stem-cell-like cell (or stemness) population because it has high aggressiveness, tumorigenicity, and self-renewal capacity [27,28] and is closely involved in distant metastasis [29,30,31]. To examine how the proportion of these cells changed, we performed a flow cytometric analysis of parental, TS, and ReA cells. To accurately define cells expressing surface markers, we used cells known not to express these markers as negative controls. MIA PaCa-2 [32], OVCAR3 [33], and MDA-MB-231 [34] cells for ESA, CD44, and CD24, respectively (Figure 2A). For isolation of the ESA^+^CD44^+^CD24^−^ population, we first gated the ESA^+^ population followed by the CD44^+^CD24^−^ population (Figure 2B). Flow cytometric analysis showed that ~4% of parental cells and ~50% of ReA cells were ESA^+^CD44^+^CD24^−^ (Figure 2C), suggesting that the stemness population is enriched in ReA cells. To determine how long the enriched ESA^+^CD44^+^CD24^−^ population was retained, we cultured ReA cells for at least 30 passages (approximately 3 months). This population remained at a level similar to passage 9 until passage 20, and decreased slightly at passage 30 (Figure 2D). These results suggest that ReA cells maintain a high stemness population for a significant period of time, even under the same culture conditions as the parental cells.

### 3.3. Cholesterol Synthesis Is Upregulated in ReA Cells Compared with Parental Cells

**Figure 3:**
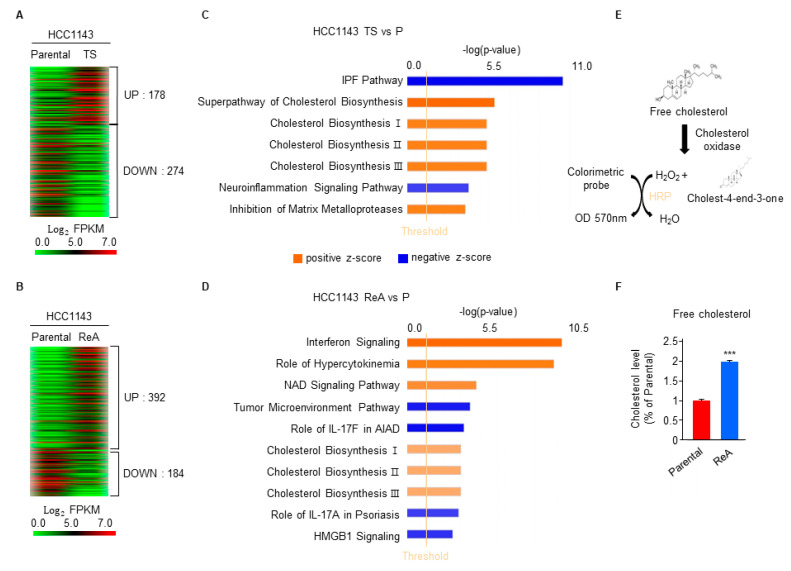
Cholesterol synthesis is upregulated in ReA cells compared with parental cells. (**A**,**B**) Differentially expressed genes (DEGs) between parental and TS-cultured cells, and parental and ReA cells were analyzed by RNA sequencing. DEGs with expression levels altered more than two-fold are shown as heatmaps produced using a Multi-Experiment Viewer. (**C**,**D**) The major signaling pathways among the DEGs were analyzed via ingenuity pathway analysis. The pathways with absolute values of z-scores of at least 2 and a −log(*p*-value) of at least 1.3 are shown. IPF—Idiopathic pulmonary fibrosis signaling pathway; cholesterol biosynthesis II—cholesterol biosynthesis II (via 24,25-dihydrolanosterol); cholesterol biosynthesis III—cholesterol biosynthesis III (via desmosterol); role of hypercytokinemia—role of hypercytokinemia/hyperchemokinemia in the pathogenesis of influenza; role of IL-17F in AIAD—role of IL-17F in allergic inflammatory airway diseases. (**E**) Colorimetric cholesterol assay principle. (**F**) Lipids in HCC1143 parental and ReA cells were extracted, and free cholesterol was measured. Relative levels of free cholesterol were represented as the mean ± standard deviation of two independent experiments (*n* = 5). *** *p* < 0.001 relative to parental cells.

RNA sequencing was performed to elucidate which signaling pathways govern TS and ReA cells. A total of 452 genes in HCC1143 TS cells and a total of 576 genes in HCC1143 ReA cells were differentially expressed more than two-fold compared with parental cells (Figure 3A,B). Core analysis using Ingenuity Pathway Analysis (IPA) software revealed that cholesterol-biosynthesis-related pathways were predominantly upregulated in TS (Figure 3C). The interferon, hypercytokinemia, NAD signaling, and cholesterol biosynthesis-related pathways were upregulated in ReA cells (Figure 3D). Downregulated pathways in TS cells included the idiopathic pulmonary fibrosis and neuroinflammation signaling pathways (Figure 3B), and downregulated pathways in ReA cells included tumor microenvironment pathways, the role of IL-17F in allergic inflammatory airway diseases, the role of IL-17A in psoriasis, and HMGB1 signaling (Figure 3D). The only commonly altered pathway in TS and ReA cells compared with parental cells was the cholesterol-synthesis-related pathway (Figure 3C,D). To determine whether the amount of cholesterol was actually altered, as predicted by RNA sequencing analysis, intracellular cholesterol levels were measured. The intracellular free-cholesterol concentration was twice as high in ReA cells compared with that of parental cells (Figure 3E,F).

### 3.4. Cholesterol Synthesis Plays an Important Role in the Malignancy of ReA Cells

**Figure 4:**
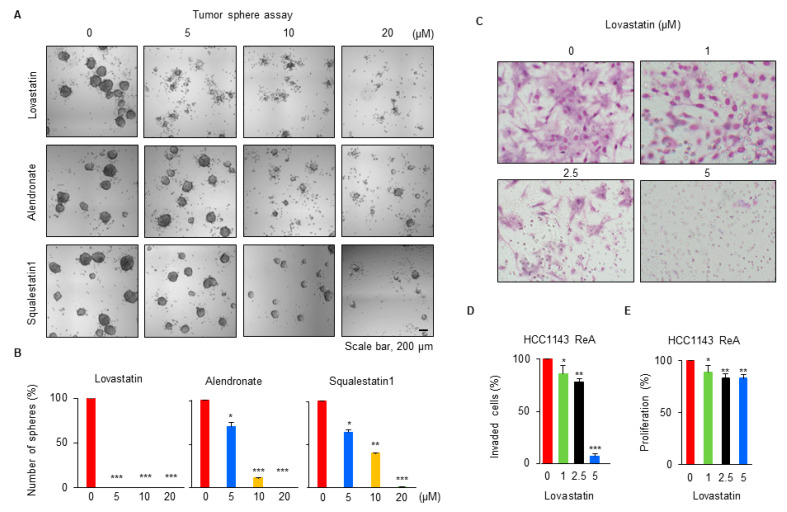
Cholesterol synthesis plays an important role in the malignancy of ReA cells. (**A**) The cholesterol biosynthesis inhibitors lovastatin, alendronate, and squalestatin1 affect TS formation. HCC1143 ReA cells were treated with the indicated concentrations of the drugs and cultured under TS-forming conditions for 7 days. Bright-field images of cells were taken with a Cytation 3 Cell Imaging Multi-Mode Reader. (**B**) The proportion of spheres was quantified as the mean ± standard deviation of three independent experiments. * *p* < 0.05, ** *p* < 0.01, *** *p* < 0.001 relative to non-treated cells. (**C**) HCC1143 ReA cells were treated with indicated concentrations of lovastatin for 48 h, and then seeded for Boyden chamber invasion assay. After 16 h, invaded cells were stained and counted. (**D**) The proportion of invaded cells was quantified as the mean ± standard deviation of three independent experiments. * *p* < 0.05, ** *p* < 0.01, *** *p* < 0.001 relative to no treat cells. (**E**) The effect of lovastatin on HCC1143 ReA cell growth under the conditions shown in Figure C was determined by SRB assay. Relative proliferative of cells was quantified as the mean ± standard deviation of three independent experiments. * *p* < 0.05, ** *p* < 0.01 relative to no treat cells.

Since ReA cells were malignant (Figure 1) and elevated in the cholesterol synthesis pathway (Figure 3), we determined whether cholesterol synthesis affects ReA cell malignancy. Treatment with cholesterol biosynthesis inhibitors, lovastatin [35], alendronate [36], and squalestatin1 [37] significantly reduced TS formation in HCC1143 ReA cells, and lovastatin was especially effective at the lowest concentration (Figure 4A,B). Lovastatin treatment almost inhibited the invasion of ReA cells at 5 μM concentration (Figure 4C,D). Treatment with the same concentration of lovastatin slightly reduced cell proliferation in the SRB assay (Figure 4E). These results suggest that the increased cholesterol synthesis pathway in ReA cells plays an important role in TS formation and invasion, not just a consequence.

### 3.5. Cholesterol Synthesis Is Important for the Invasion of Breast Cancer Cells

**Figure 5:**
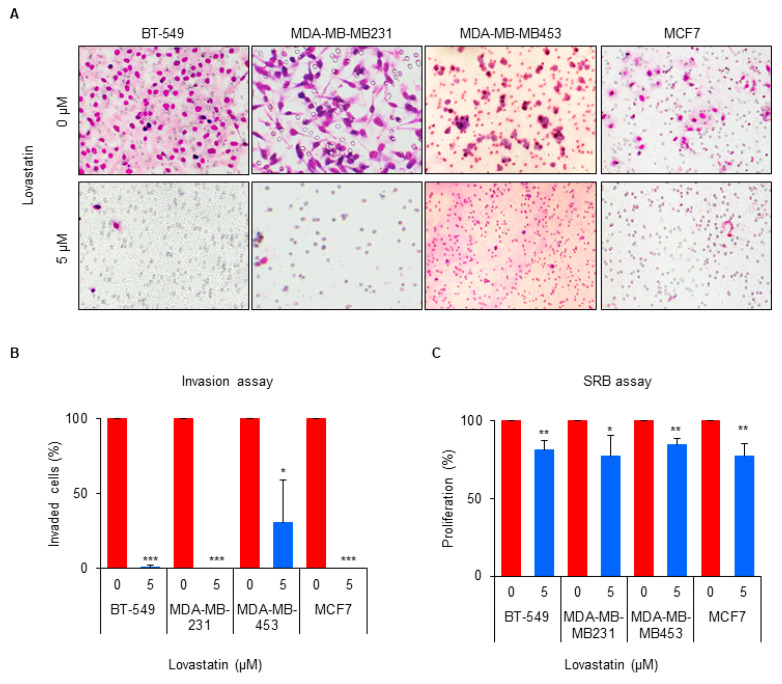
Cholesterol synthesis is important for the invasion of breast cancer cells. (**A**) BT-549, MDA-MB-231, MDA-MB-453, and MCF7 cells were treated with the 5 μM of lovastatin for 48 h, and then seeded for Boyden chamber invasion assay. After 16 h, invaded cells were stained and counted. (**B**) The proportion of invaded cells was quantified as the mean ± standard deviation of three independent experiments. * *p* < 0.05, *** *p* < 0.001 relative to no treat cells. (**C**) The effect of lovastatin on breast cancer cell growth under the conditions shown in Figure A was determined by SRB assay. The relative proliferation of cells was quantified as the mean ± standard deviation of three independent experiments. * *p* < 0.05, ** *p* < 0.01 relative to non-treated cells.

Next, we determined whether the cholesterol synthesis pathway is important for invasion not only in HCC1143 ReA cells, but also in other breast cancer cell lines. Lovastatin treatment significantly inhibited the invasion of BT-549, MDA-MB-231, MDA-MB-453, and MCF7 parent cells at 5 μM (Figure 5A,B). Under the same conditions, lovastatin slightly reduced the proliferation in the SRB assay (Figure 5C). Overall, these results suggest that cholesterol synthesis is important for distant metastasis of various breast cancer cells.

## 4. Discussion

Although the 5-year survival rate is high, after 5 years, a significant number of breast cancer patients recur as distant metastases, and the prognosis of these patients is extremely poor [38]. In order to reduce breast cancer mortality, it is important to suppress distant metastasis, but the important pathways in the process of distant metastasis are largely unknown. Previous studies have suggested a possible association between cholesterol and distant metastasis in breast cancer. Obesity is linked with a greater risk of breast cancer recurrence [39,40], particularly with distant recurrence [41], and the majority of breast cancer patients with distant recurrence are obese [42]. Since elevated cholesterol is significantly associated with obesity [43,44], high cholesterol levels may be associated with breast cancer distant metastasis in humans. In a mouse model of breast cancer, which was fed a high-fat diet, cholesterol levels and distant metastases to lung tissue were increased [13]. Very-low-density lipoprotein (VLDL) cholesterol has increased breast cancer cell migration and invasion in vitro and metastasis in a mouse tail vein injection model [45]. Cholesterol synthesis has been increased in the mammosphere, and inhibition of cholesterol synthesis reduced mammosphere formation in ER breast cancer [17]. In addition, cholesterol synthesis inhibitors have reduced immigration and invasion of breast cancer cells [46,47,48,49]. In this study, we found that cholesterol synthesis pathways were increased in TSs and ReA cells, mimicking CTCs and cancer metastases. We also found that inhibition of the cholesterol synthesis pathway reduced TS formation and breast cancer cell metastasis. These findings suggest the potential to modulate breast cancer distant metastasis by inhibiting cholesterol synthesis.

Cancer stem-cell-like cells (or cancer cells with stemness) have been linked with metastasis of breast cancer [3,50], and breast cancer cells with stemness have been associated with cholesterol synthesis [17,51,52]. Mammosphere isolated from patient-derived xenograft tumors from ER- breast cancers have been enriched for cancer stem cells, and cholesterol synthesis is important for mammosphere formation [17]. Squalene epoxidase (SQLE) has increased cholesterol synthesis and intracellular cholesterol has been involved in mammosphere maintenance [52]. In this study, the ESA^+^CD44^+^CD24^−^ population of breast cancer, which has been widely used as a marker for breast cancer cells with stemness [27], was enriched in ReA cells as well as TSs (Figure 2). The only pathway commonly altered in TS and ReA cells compared to parental cells was cholesterol synthesis by RNA sequencing analysis (Figure 3). The amount of intracellular cholesterol was increased in ReA cells (Figure 3F), and suppression of cholesterol synthesis reduced TS formation and metastasis of ReA cells (Figure 4). These data suggest that cholesterol synthesis is important for breast cancer cells with stemness, thereby playing an important role in distant metastasis.

**Figure 6:**
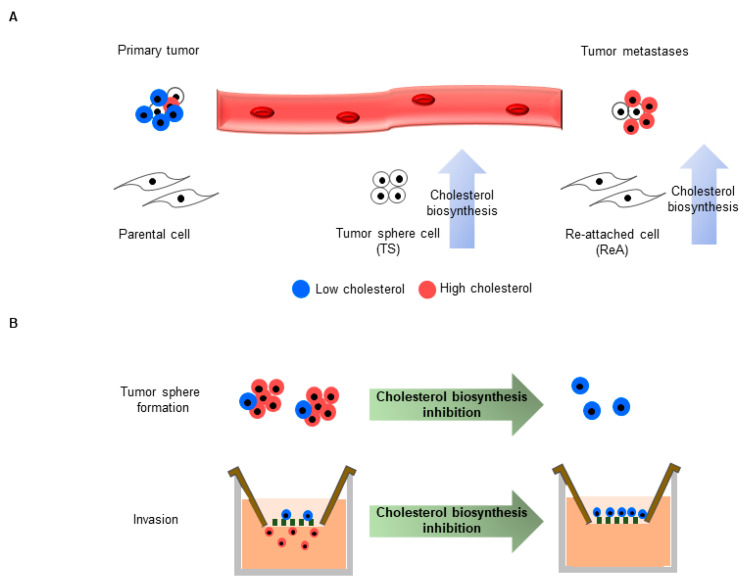
A schematic model showing the relationship between cholesterol synthesis and distant metastasis. (**A**) During distant metastasis of breast cancer, the primary tumor metastasizes via CTCs. Cholesterol synthesis is commonly increased in CTC-mimicking TS and tumor metastases-mimicking ReA cells. (**B**) Inhibition of cholesterol synthesis reduces tumor sphere formation and invasion ability.

Overall, we found that the cholesterol biosynthesis pathway was increased not only in breast cancer TSs, but also in ReA cells, and that pharmacological inhibition reduced TS formation capacity and invasion of ReA cells (Figure 6). Considering the previous reports that cholesterol metabolism is important in various cancer stemness [18,53,54] and metastasis [55], cholesterol metabolism may be important in various cancers as well as breast cancer. In addition, the results of this study will help us understand the relationship between obesity and cancer and develop strategies to prevent breast cancer distant metastasis.

## 5. Conclusions

Our findings show that breast cancer cells reattached after tumor sphere (TS) culture, in part mimicking cancer distant metastasis, are malignant compared to parental cells and are rich in cancer stem-cell populations. The cholesterol biosynthesis was the predominant pathway upregulated in both TS and reattached cells. Pharmacological inhibition of cholesterol synthesis reduced TS formation and invasion ability. These results suggest that the cholesterol synthesis pathway plays an important role in breast cancer distant metastasis.

## Data Availability

The RNA-seq data were deposited in the Gene Expression Omnibus database under the accession number GSE203506.

## Abbreviations

P—parental; 2D—two-dimensional; TS—tumor sphere; ReA—reattached; CTCs—circulating tumor cells; SRB—sulforhodamine B—DEGs—differentially expressed genes; IPA—ingenuity pathway analysis.
